# Immersive Virtual Reality Assessment in Multiple Sclerosis: Patient-Reported Experience and Correlates

**DOI:** 10.3390/brainsci16040433

**Published:** 2026-04-21

**Authors:** Anikó Vágó, Anne Geßner, Maximilian Hartmann, Heidi Stölzer-Hutsch, Katrin Trentzsch, Dirk Schriefer, Tjalf Ziemssen

**Affiliations:** University Hospital Carl Gustav Carus, TU Dresden, Center of Clinical Neuroscience, Neurological Clinic, Dresden, and Centre for Tactile Internet with Human-in-the-Loop (CeTI), 01309 Dresden, Germany; ani.v@web.de (A.V.); anne.gessner@ukdd.de (A.G.); maximilian.hartmann@ukdd.de (M.H.); heidi.stoelzer-hutsch@ukdd.de (H.S.-H.); katrin.trentzsch@ukdd.de (K.T.); dirk.schriefer@ukdd.de (D.S.)

**Keywords:** virtual reality, multiple sclerosis, patient-reported experience, upper-limb assessment, PREMs

## Abstract

**Background:** Immersive virtual reality (VR) has emerged as a promising tool for standardized, engaging assessment of motor and cognitive function in people with multiple sclerosis (pwMS). However, patient-reported experiences with immersive VR tasks have not been systematically evaluated. **Objective:** To characterize patient-reported experience measures (PREMs) after a multidomain immersive VR task and explore relationships with clinical characteristics, therapeutic history, and task performance. **Methods:** In this prospective cross-sectional study, participants completed a seated immersive VR task comprising six upper-limb tasks with motor and cognitive components. Patient experience was evaluated immediately afterward using a PREM questionnaire. Upper-limb activity limitations were assessed with the Arm Function in Multiple Sclerosis Questionnaire (AMSQ). **Results:** A total of 129 pwMS (EDSS 3.5–8.0) participated. Median PREM item scores ranged from 1.0 to 2.0 (scale 0–10), indicating an overall positive experience. Over 80% rated staff support as excellent; more than half perceived the assessment as safe, comfortable, and appropriately timed. An amount of 40.3% of pwMS wished to use VR tasks more often than once per year. PwMS receiving upper-limb physiotherapy or occupational therapy reported greater perceived difficulty than those without therapy. In exploratory analyses, higher perceived difficulty and a preference for less frequent VR use were associated with higher EDSS (r = 0.208 and 0.200) and ambulation scores (r = 0.215 and 0.195). Difficulty ratings were also related to pyramidal (r = 0.188) and sensory (r = 0.174) impairments. **Conclusions:** PwMS reported a positive overall experience with the immersive VR tasks. Further studies should evaluate the suitability and validity of this approach compared with conventional assessments.

## 1. Introduction

Multiple sclerosis (MS) is a chronic inflammatory autoimmune disease that leads to the demyelination of nerve cells in the brain and spinal cord [1]. MS typically affects young adults and can cause physical and cognitive disability, impacting their quality of life and ability to participate in daily activities [2,3]. Upper-limb dysfunction is a common symptom, affecting around 50–65% of people with multiple sclerosis (pwMS) [2]. Although both the upper and lower extremities are equally affected, the lower limb usually receives more attention than the upper limb [2,4].

Virtual reality (VR) is increasingly used in upper-limb neurorehabilitation and offers an innovative, potentially effective approach for pwMS [5,6,7,8]. As a computer-generated 3D environment, VR can be delivered in non-immersive (e.g., monitor-based), semi-immersive (e.g., large screens), and fully immersive formats (head-mounted displays) [9]. Adoption of immersive VR in healthcare has accelerated in recent years and may provide cost-effective gains in neurorehabilitation outcomes [10,11,12].

A key advantage of VR-supported functional assessment is the ability to engage patients in ecologically valid, simulated everyday situations without the influence of a therapist’s presence, allowing more naturalistic observation of movement and behavior. VR systems also capture detailed temporal–spatial kinematics and enable automated analysis, remote delivery, and home-based monitoring, supporting comprehensive functional profiling and continuous symptom tracking [13]. By embedding functional, goal-oriented, and motivational elements, VR can personalize therapy content while simultaneously documenting motor and cognitive impairments relevant to treatment planning [9,14,15].

Routine neurological status in MS is commonly summarized by the Expanded Disability Status Scale (EDSS) [16], but several studies indicate that traditional assessments may lack sensitivity to subthreshold deficits and latent disease burden compared with more challenging, performance-based measures [17,18,19].

Emerging evidence suggests immersive VR may offer a sensitive assessment of cognitive and motor function. In pwMS, VR-based evaluations have captured cognitive and behavioral performance within ecologically valid scenarios and detected subtle impairments beyond conventional tests [20]. Although some data come from interventional contexts, VR tasks that simulate everyday demands (e.g., virtual shopping) appear to engage executive functions and instrumental activities of daily living in ways that translate to standardized outcomes [21]. Moreover, VR adaptations of established dexterity tests (e.g., Box and Block Test, Nine-Hole Peg Test) have shown promising validity in other neurological conditions such as Parkinson’s disease and stroke [13,22,23,24,25]. Building on this groundwork, the VR system used here delivers six integrated tasks—attention, range of motion, reaction, holding, accuracy, and memory—enabling simultaneous assessment of motor and cognitive domains.

Despite this potential, the use of immersive VR for functional assessment of upper-limb and cognitive performance has not been systematically evaluated in pwMS, and patient experience with such assessments remains underexplored. Understanding patient-reported experience is essential for clinical implementation, adherence, and identification of barriers to technology-supported assessment. Prior PREM studies in pwMS have focused on diagnosis, treatment, and communication with healthcare providers, and early work on VR therapies suggests that usability, comfort, and side effects shape patient acceptance [26].

The primary aim of this exploratory study was to describe patient-reported experiences (PREMs) of participating in a multidomain immersive VR task. A second aim was to describe first exploratory associations of PREM outcomes with disability level, upper-limb activity (AMSQ), demographics, therapeutic history, and VR task performance.

## 2. Materials and Methods

### 2.1. Participants

We conducted a prospective exploratory cross-sectional study at the Multiple Sclerosis Center, Center of Clinical Neuroscience, Department of Neurology, University Hospital Carl Gustav Carus, Dresden, Germany. Between July 2023 and January 2024, we consecutively recruited 129 people with MS (pwMS).

Inclusion criteria: (1) MS diagnosed according to the McDonald criteria; (2) age ≥ 18 years; (3) EDSS ≤ 8.5; (4) ability to understand and follow simple instructions; and (5) British Medical Research Council (BMRC) grade ≥ 3 in the upper extremities.

Exclusion criterion: visual or hearing impairment that would preclude adequate perception of VR stimuli.

The study was approved by the local ethics committee (BO-EK-446102023). All participants provided written informed consent.

### 2.2. Virtual Reality Tasks

VR tasks were performed with a fully immersive VR system (CUREO^®^3, version 3.0xH1; CUREosity GmbH, Düsseldorf, Germany) comprising a head-mounted display (Oculus Quest), two handheld controllers, and a tablet. Before testing, trained staff standardized the procedure and familiarized participants with the equipment and virtual environment. Three trained raters administered all assessments. For safety, participants were seated at a table while wearing the headset.

The VR tasks comprised six upper-limb tasks administered in a fixed order:

- Attention: Follow a moving virtual butterfly with your gaze.
- Range of motion: Move both arms through the three axes of motion.
- Reaction: Reach to press a virtual button as quickly as possible.
- Holding: Maintain both arms at shoulder height for 30 s.
- Accuracy: Trace predefined shapes as precisely as possible.
- Memory: Memorize and reproduce sequences of buttons with increasing length.

**Figure 1:**
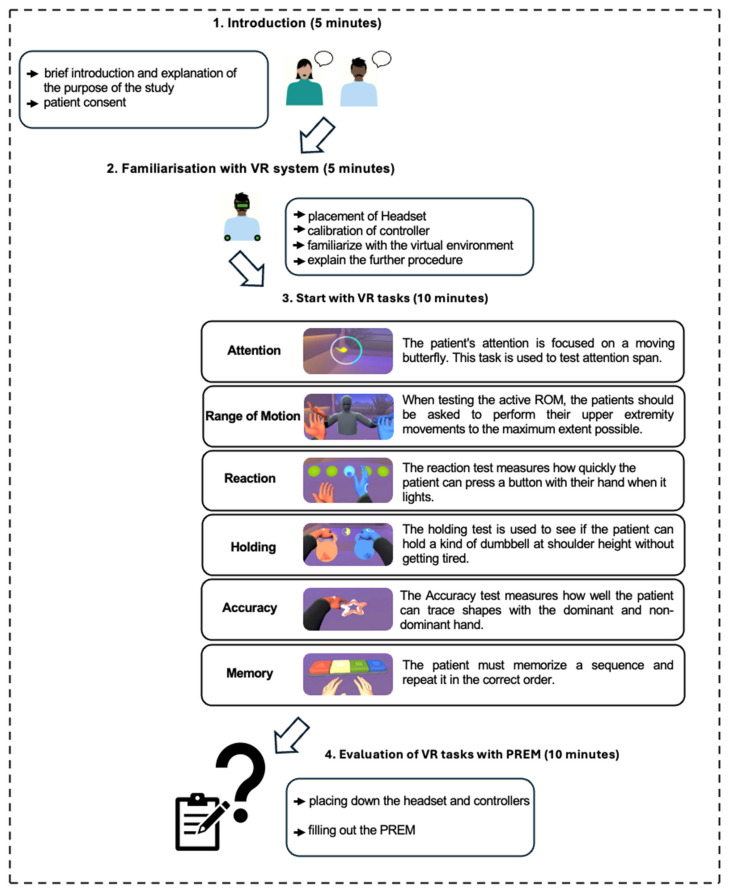
Description of the VR tasks procedure. The VR tasks used in the study (own depiction). Abbreviations: ROM = Range of Motion; PREM = Patient-Reported Experience Measures; VR = virtual reality.

Each task yielded a system-automated score from 0 (limited function) to 100 (unlimited function). These scores were generated automatically by the device software from task-specific performance parameters recorded during the VR tasks. However, as noted in the manufacturer’s instructions for use, these parameters are approximate and should not be interpreted as direct measures of clinical status. Detailed scoring information is provided in Appendix A, Table A1 and in the device instructions for use (from p. 115 to 116). Figure 1 summarizes the test workflow and task set.

### 2.3. Questionnaires

#### 2.3.1. PREM Questionnaire

Participants completed a paper-based PREM immediately after the VR tasks under staff supervision (Figure 1). The questionnaire was used as an adapted instrument and comprised ten items covering ease of use, perceived strain, comfort (2 items), usefulness and relevance (3 items), staff support (1 item), and appropriateness of session time and frequency (2 items) (Table 1). Items were rated on an 11-point Likert scale from 0 (positive) to 10 (negative). The questionnaire also asked whether participants had received physiotherapy or occupational therapy for upper-limb motor deficits and whether they had prior VR experience (yes/no). Table 1 lists all items and scoring interpretations.

#### 2.3.2. Expanded Disability Status Scale (EDSS)

To characterize neurological clinical status, we used the Expanded Disability Status Scale (EDSS). Neurostatus-certified raters assessed each participant, generating Functional System Scores (FSSs) across motor, sensory, visual, cerebellar, brainstem, bowel/bladder, and cerebral (mental) domains [16]. The EDSS is the most widely used disability scale in MS and is well established in clinical practice [28].

#### 2.3.3. Arm Function in Multiple Sclerosis Questionnaire (ASMQ)

The German Arm Function in Multiple Sclerosis Questionnaire (AMSQ) is a valid, reliable measure of upper-limb manual dexterity and its impact on activities of daily living and quality of life in German-speaking pwMS [29]. It comprises 31 items rated on a 6-point Likert scale from 1 (“not at all”) to 6 (“can no longer”). Total scores range from 31 to 186, with higher scores indicating greater limitation [30].

### 2.4. Statistical Analysis

Analyses were performed in IBM SPSS Statistics for Windows, version 28 (IBM Corp., Armonk, NY, USA). Distributions were inspected using histograms and Q–Q plots; normality was additionally assessed with the Shapiro–Wilk test. Continuous variables are reported as mean ± SD or median (IQR), as appropriate; categorical variables are reported as n (%). PREM items are summarized descriptively as median (IQR) and/or % of participants. The AMSQ total score was converted to percentages and categorized as unrestricted (0%), moderately restricted (1–50%), and severely restricted (51–100%). For subgroup analyses of patient experience, pwMS were stratified by prior physiotherapy (yes/no), occupational therapy (yes/no), and VR experience (yes/no). Between-group differences in PREM items were tested with the nonparametric Mann–Whitney U test. We applied a Bonferroni–Holm correction to counteract the problem of multiple comparisons. The exploratory relationship between each of the PREM items and sex, age, clinical outcomes, AMSQ, and VR task scores was examined using Spearman’s rank correlation. Correlation magnitudes were interpreted as small (|r| ≈ 0.10), moderate (|r| ≈ 0.30), and large (|r| ≥ 0.50). Two-tailed *p* values < 0.05 were considered statistically significant.

## 3. Results

### 3.1. Participants

**Figure 2:**
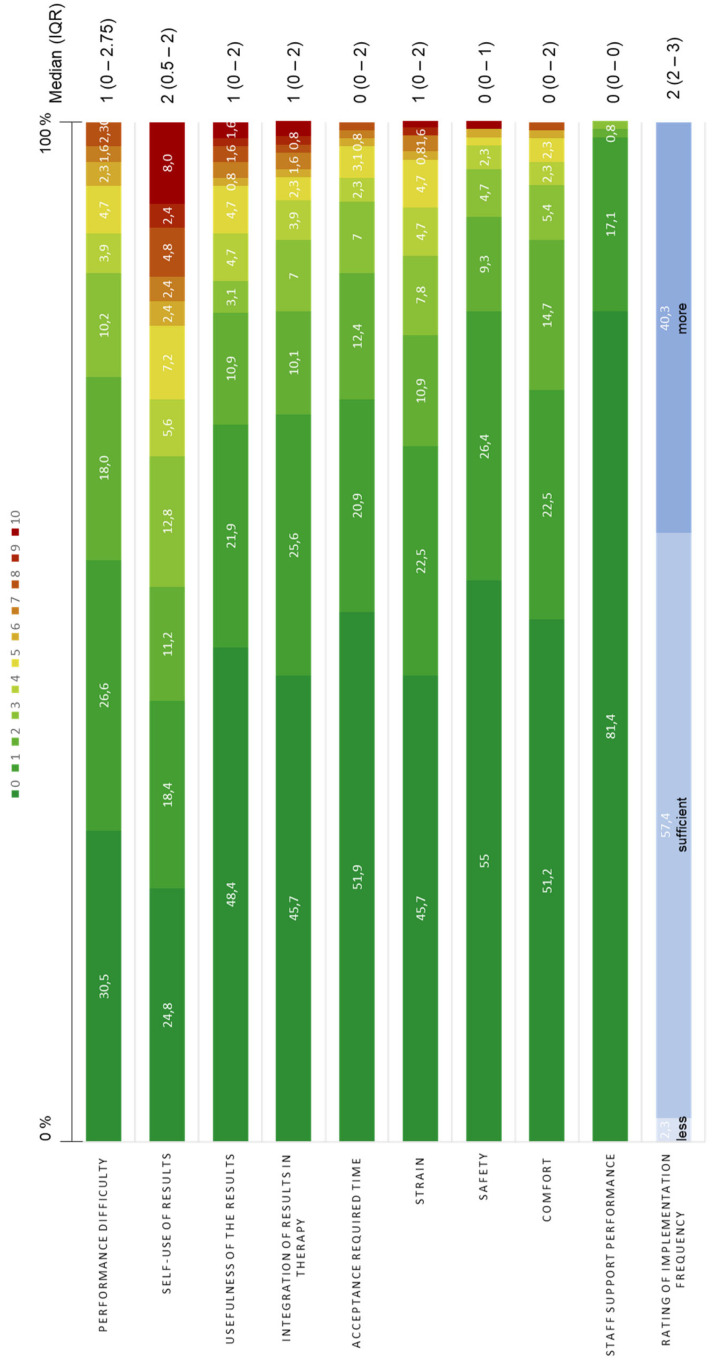
Response distribution in percent (%) and median (IQR) of the patient experience for the PREM items of the VR tasks in pwMS (*N* = 129). The colors represent the response scale from 0 (green) to 10 (red) of the patient survey PREM questionnaires. Abbreviations: IQR = Interquartile Range, VR = virtual reality.

A total of 129 pwMS completed the VR tasks and were included in the analysis (Figure 2), supporting the feasibility of the procedure. Median EDSS was 3.5 (IQR 2.25–6.0), indicating moderate to substantial ambulatory disability. Based on the AMSQ, 21.7% reported unrestricted upper-limb function. Overall, 51.2% were receiving physiotherapy for upper-limb impairments, 27.2% occupational therapy, and 18.6% reported prior VR experience. Descriptive characteristics, including AMSQ data, are summarized in Table 2.

### 3.2. PREM Questionnaire

The PREM questionnaire was completed by 125 of 129 pwMS. Item medians ranged from 1.0 to 2.0 on an 11-point scale (0 = positive, 10 = negative), indicating a generally positive experience (Figure 2). Most participants (81%) rated staff support as very good (median 0, IQR 0–0). Over half perceived the assessment as safe (55%; median 0, IQR 0–1), comfortable (51%; median 0, IQR 0–2), and appropriately timed (52%; median 0, IQR 0–2). Only 8% reported that the results were not useful for their own review. Overall, the assessment was rated as easy (median 1, IQR 0–2.75) and not very exhausting (median 1, IQR 0–2). A single implementation per year was considered sufficient by 57.4% of pwMS. Figure 2 displays the percentage distribution of PREM responses as stacked bar charts. Median PREM scores are provided in Appendix A, Table A2.

### 3.3. PREM Outcome of VR Tasks According to Physiotherapy and Occupational Therapy

**Figure A1:**
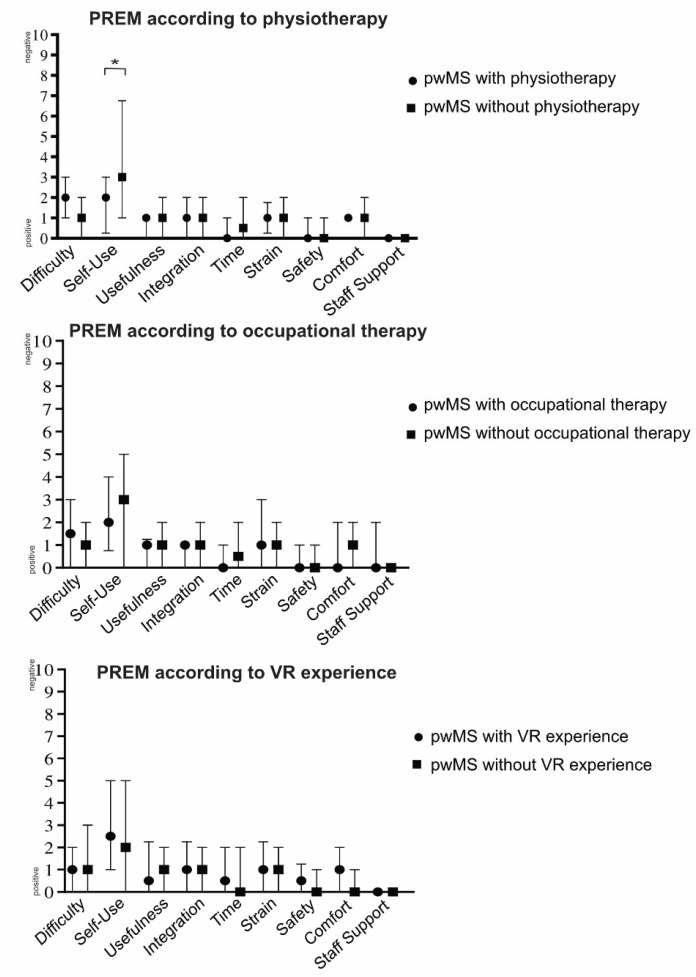
Patient experience (median, IQR) according to physiotherapy, occupational therapy and VR experience. * = significant difference (*p* < 0.05) according to Mann–Whitney U Test. Abbreviations: pwMS = people with Multiple Sclerosis, PREM = Patient-Experience Measures, VR = virtual reality.

PwMS receiving upper-limb physiotherapy or occupational therapy reported greater perceived difficulty than those without therapy. Participants in physiotherapy (N = 66) also reported greater self-use of the VR tasks results than those without physiotherapy (N = 63) (*p* = 0.013). Those in physiotherapy more often preferred assessments more frequently than once per year, whereas those without physiotherapy considered an annual assessment sufficient (*p* = 0.09). Patient experience (median, IQR) according to physiotherapy, occupational therapy and VR experience is shown in Appendix A, Figure A1.

### 3.4. Association Between PREM and VR Tasks, Clinical Outcomes, Age, Sex and AMSQ

Overall, fewer and smaller significant correlations between PREM items and sex, age, clinical outcomes, AMSQ, and VR task were found. Higher perceived difficulty and preference for more frequent VR tasks showed small associations with higher ambulation (r = 0.215 and 0.195) and EDSS scores (r = 0.208 and 0.200). Greater abnormalities in the pyramidal (r = 0.188) and sensory (r = 0.174) functional systems seemed to be associated with greater perceived difficulty. Higher age tended to be associated with higher perceived strain (r = 0.257). Greater upper-limb activity limitation on the AMSQ was related to higher perceived difficulty (r = 0.224) and strain (r = 0.272). In addition, greater perceived difficulty was associated with poorer performance on VR active range of motion (aROM), holding, and memory. Details of correlations are summarized in Appendix A, Table A4. Median PREM scores by sex, age, AMSQ, and EDSS subgroups are provided in Appendix A, Table A2. Median VR scores are provided in Appendix A, Table A3.

## 4. Discussion

This prospective exploratory cross-sectional study is, to our knowledge, the first to evaluate patient-reported experiences with immersive VR tasks in pwMS.

Overall, median PREM item scores ranged from 1.0 to 2.0 (scale 0–10), indicating an overall positive experience. PwMS reported high levels of comfort, safety, usefulness, and perceived relevance. Over 80% rated staff support as excellent; more than 50% perceived the assessment as safe, comfortable, and appropriately timed. These findings align with prior work showing that VR applications can enhance motivation, compliance, and repetition compared with conventional approaches [31,32], with satisfaction positively linked to adherence [5]. In our cohort, 40.3% wished to use VR tasks more often than once per year. These observations are further supported by findings from Geßner et al. who reported that pwMS rated a sensor-based jump assessment highly in terms of comfort, perceived usefulness, and therapeutic relevance, underscoring that digital functional testing is generally well accepted when designed to be intuitive and clinically meaningful [27].

Disability levels appeared to be related to patient experience: lower EDSS and better ambulation were associated with less difficulty and a greater desire for regular use, whereas pwMS with EDSS 3.5–8 still reported positive experiences overall—consistent with reports of good acceptance among patients with higher disability. Exploratory analysis suggested a trend that older pwMS perceived greater strain, suggesting that novel, cognitively demanding interfaces may require simplified or more intuitive task designs for older users (e.g., hand-tracking modes) [33]. Similar patterns were reported in the long-term MSPT study, where older and disabled people had a harder time with digital assessments. This shows that it is important to design VR tasks that are easy for these groups to use [34].

Upper-limb activity limitations (AMSQ) were associated with higher perceived difficulty and strain, suggesting that motor deficits shape user experience. Similar tendencies, though largely nonsignificant, were observed in those receiving physiotherapy or occupational therapy for upper-limb deficits. Notably, prior VR experience did not affect PREM outcomes, underscoring the user-friendliness of the assessment.

Evidence also suggests that VR environments can sensitively probe cognitive domains—often with greater ecological validity than traditional tests—addressing real-world challenges such as attention and memory that constrain daily life [13,20]. Nevertheless, technical hurdles (e.g., system reliability, setup time) still limit widespread adoption [24]. If addressed, VR could enable simultaneous, parallel assessment of motor and cognitive functions in realistic contexts (e.g., cooking performance relates to cognitive impairment in pwMS) [35], supporting immediate, tailored therapeutic decisions.

Our PREM questionnaire is a self-developed questionnaire and has been used intensively in a similar form to evaluate patient experience with the Dresden protocol for multidimensional gait analysis in a study by Scholz et al. and with jump assessment in a study by Geßner et al. [27,36,37]. For the present study, the questionnaire was adapted for the Jump VR tasks. Based on their use in previous studies, the selection and adaptation of the items were guided by the areas of patient experience relevant to digital health tools, as described by the Agency for Healthcare Research and Quality [38]. The staff supervision during PREM completion may have biased responses toward positive ratings (social desirability) [39], and the cross-sectional design precludes causal inference.

Fewer correlations between PREM items and VR task performance were significant but small, with accuracy showing the broadest associations—particularly with perceived safety and integration into therapy. These effect sizes warrant cautious interpretation. Overall, the correlation analyses should be regarded as exploratory and hypothesis-generating. Due to the exploratory nature of this study, we did not adjust for multiple comparisons in correlations, and results should be interpreted cautiously [40].

A further key limitation of this study is that the six-task VR battery is a novel tool and not yet a validated assessment tool. Although it is designed to capture multiple motor and cognitive domains (e.g., attention, range of motion, reaction time, holding, accuracy, and memory), its ability to accurately and reliably detect impairments in pwMS has not been established. Future studies are needed to systematically validate the performance-based VR tasks, including comparisons with established clinical and functional assessments such as the Box and Block Test and the Nine-Hole Peg Test. VR tasks could deliver challenging yet safe tasks while standardizing protocols and documentation, enabling targeted functional testing and automated capture of performance data [14]. Given the complexity of MS symptoms, such technologies may help identify impairments earlier and visualize limitations in ecologically valid, everyday-like scenarios to guide treatment planning [41,42,43]. Beyond experience, several VR tasks (aROM, reaction, accuracy, and holding) may offer clinically meaningful insight into everyday upper-limb function and intervention targets, and could serve as pragmatic indicators of strength, stability, and therapy response [44,45]. Consistent with this, Trentzsch et al. showed that digital, instrumented assessments can reveal subtle impairments not evident in conventional scales, underscoring the added value of objective measurement in regarding specific pathological gait profiles [46].

A further limitation is that the VR scores are proprietary, system-derived parameters whose clinical interpretability and clinimetric properties have not yet been fully established in pwMS. Future studies should validate these VR-derived scores against established assessment tools and examine their reliability, responsiveness, interpretability, and potential clinical utility in pwMS.

We did not systematically evaluate side effects; however, cybersickness can occur in immersive VR (e.g., dizziness, disorientation, blurred vision, nausea) [47]. Although tolerability is generally acceptable for diagnostic and rehabilitation purposes in pwMS, future work should incorporate standardized protocols to screen for individual susceptibility and to quantify objective and perceived effects to maximize safety and comfort.

In summary, this initial exploratory analysis suggests that immersive VR tasks may be feasible and well accepted across a broad disability spectrum in pwMS. With continued technical refinement and clinimetric validation, VR-based ecologically valid assessments may complement routine care by capturing subtle motor and cognitive impairments and informing individualized therapy.

## 5. Conclusions

This study suggests that pwMS showed a positive experience with the VR tasks, reporting high levels of comfort, safety, usefulness, and perceived integration into care. VR task performance, especially accuracy, is related to more positive patient experience ratings. These findings support the feasibility and acceptability of immersive VR for multidomain functional evaluation in MS. In order to provide a more comprehensive analysis, prospective validation of the VR tasks against established assessments and evaluations of clinimetric properties is needed.

## Figures and Tables

**Table 1:** Items of the PREM questionnaire with interpretation scale.

	PREM Item	PREM Questions	Interpretation Scale 0–10
1	Difficulty with VR tasks	How easy or difficult would you rate the VR tasks?	0 = easy; 10 = difficult
2	Self-use of results	To what extent do you use the results for your own review?	0 = always; 10 = not at all
3	Usefulness of the results	How useful do you think incorporating the results into your progress monitoring is?	0 = useful; 10 = not useful
4	Integration of results in therapy	How convinced are you that the results will be used for your disease progression?	0 = convinced; 10 = not at all convinced
5	Acceptance required time	How do you rate the time required to perform the VR tasks?	0 = appropriate; 10 = too time-consuming
6	Strain VR tasks	How exhausting do you experience the VR tasks?	0 = not exhausting; 10 = exhausting
7	Safety VR tasks	How do you rate the safety of the VR tasks?	0 = safe; 10 = unsafe
8	Comfort VR tasks	How do you rate the comfort of the VR tasks?	0 = comfortable; 10 = very uncomfortable
9	Staff support performance	How do you rate the support and explanation provided by the study staff?	0 = very good; 10 = bad
10	Rating of implementation frequency	Do you think the VR tasks once a year are sufficient (if no relapses occur)?	a. less frequent (1) b. sufficient (2) c. more frequent (3)

Abbreviations: PREM = Patient-Reported Experience Measures, VR = virtual reality. The structure of this table is consistent with our previous publication [27].

**Table 2:** Characteristics of the study population (*N* = 129). Data presented as mean (±standard deviation) unless specified otherwise.

		pwMS (*N* = 129)
Age (years)		48.78 (±12.20)
Females N (%)		65 (50.4%)
BMI		25.57 (±5.22)
AMSQ (*N* = 120)		51.19 (±27.51)
	Unrestricted *N* (0%)	28 (21.7%)
	Moderately restricted *N* (0–50%)	88 (68.2%)
	Severely restricted *N* (50–100%)	4 (3.1%)
Wheelchair *N* (%)		16 (12.4%)
Disease Duration (years) (*N* = 110)		12.49 (±9.31)
MS Type *N* (%)		
	RRMS	100 (77.5%)
	PPMS	14 (10.1%)
	SPMS	15 (11.6%)
EDSS	Median (IQR)	3.5 (2.25–6.0)
	Visual FSS	1.0 (0–2.0)
	Brainstem FSS	1.0 (1.0–2.0)
	Pyramidal FSS	3.0 (1.0–3.0)
	Cerebellar FSS	2.0 (1.0–2.0)
	Sensory FSS	2.0 (1.0–2.0)
	Ambulation (N = 128)	1.0 (0–2.0)
Physiotherapy *N* (%)		66 (51.2%)
Occupational Therapy *N* (%) VR Experience		35 (27.2%) 24 (18.6%)

Abbreviations: pwMS = people with Multiple Sclerosis; BMI = Body Mass Index; AMSQ = Arm Function in Multiple Sclerosis Questionnaire; RRMS = Relapsing–Remitting Multiple Sclerosis; PPMS = Primary Progressive Multiple Sclerosis; SPMS = Secondary Progressive Multiple Sclerosis; EDSS = Expanded Disability Status Scale; FSS = Functional System Score; IQR = Interquartile Range; *N* = total number; VR = virtual reality.

## Data Availability

The datasets generated and/or analyzed during the current study are not publicly available due to institutional and ethical restrictions, but are available from the corresponding author on reasonable request.

## Appendix A

**Table A1:** Description of the scores from the VR tasks.

VR Task	Definition of the Score
Attention	100 points are achieved when full concentration is maintained on the butterfly throughout the entire task. For each second the gaze deviates from the butterfly, points are deducted. Half the concentration time results in half the points.
Active Range of Motion	100 points are achieved if the active range of motion (estimated from the player’s height) is almost fully reached. For example, reaching 60% of this range results in 60 points.
Holding	100 points are achieved if the patient maintains an average deviation of <2 from shoulder height throughout the 15-s duration. In the range between 2 and 4, the patient loses percentage points linearly until, with an average deviation of 4 or more, 0% is reached.
Reaction Time	100 points correspond to a good value that unimpaired subjects can achieve. 0 points are awarded for a reaction time four times longer.
Accuracy	100 points are achieved if the line is traced very well (green line) to acceptably (yellow line). An error is produced if the patient deviates significantly from the predefined line, indicated by a red line in the diagram. Each error results in a 5-point deduction.
Memory	The task is based on rounds until a sequence of 15 buttons is reached. 100 points are achieved if all rounds are completed successfully. For each round not completed, 14 points are deducted.

Abbreviations: VR = virtual reality, EDSS = Expanded Disability Status Scale, AMSQ = Arm Function in Multiple Sclerosis Questionnaire, A = significant difference (*p* < 0.05).

**Table A2:** Detailed description of the PREM items overall and regarding sex, age, EDSS and AMSQ (median (IQR)).

PREM		Sex		Age			EDSS				AMSQ		
	Overall (N =129)	Female N = 65	Male N = 64	Young N = 15	Middle-Age N = 84	Old N = 30	EDSS 0–1.5 N = 25	EDSS 2–3 N = 32	EDSS 3.5–5.5 N = 40	EDSS 6–8 N = 32	Unrestricted N = 28	Moderately Restricted N = 87	Severely Restricted N = 4
Performance difficulty VR task	1 (0–2.75)	1 (0–2)	1 (0–3)	0 (0–0.75)	1 (0–2)	1 (0–3)	1 (0–2)	1 (0–2)	1 (0–3)	2 (1–4)	1 (0–2)	1 (0–2)	3 (1.25–4.75)
Self-use of results	2 (0.5–5)	2 (0–4.25)	2 (1–5)	1 (0–2.75)	2 (0.5–5)	2 (1–5)	3 (1–5)	1 (0–4.75)	2 (1–5)	2 (1–4)	3 (0.25–5)	2 (1–5)	2 (0.5–5.75)
Usefulness of the results	1 (0–2)	0 (0–0)	1 (0–2)	0 (0–0)	1 (0–2)	1 (0–2)	2 (0–3.5)	0 (0–1)	1 (0–1.25)	1 (0–2)	1 (0–3.75)	1 (0–2)	1 (0–4.25)
Integration of results in therapy	1 (0–2)	1 (0–1.5)	1 (0–2)	1 (0.25–1)	1 (0–2)	0 (0–2)	1 (0–2)	0.5 (0–1.75)	1 (0–2)	0 (0–1)	1 (0–2)	1 (0–2)	0.5 (0–3.25)
Acceptance required time	0 (0–2)	0 (0–1)	1 (0–2)	0 (0–0)	1 (0–2)	0 (0–1)	0 (0–2)	0 (0–1)	1 (0–2)	1 (0–2)	0 (0–1)	1 (0–2)	0 (0–0.75)
Strain VR task	1 (0–2)	0 (0–1.5)	1 (0–3)	1 (0–2)	1 (0–2)	1 (0–2)	0 (0–1.5)	1 (0–2)	1 (0–2)	1 (0–3)	0 (0–0)	1 (0–3)	0.5 (0–4)
Safety VR task	0 (0–1)	0 (0–1)	0 (0–1)	0 (0–0)	0 (0–1)	0 (0–1)	0 (0–1)	0 (0–1)	1 (0–2)	0 (0–1)	0 (0–1)	0.5 (0–1)	0.5 (0–4)
Comfort VR task	0 (0–2)	0 (0–1)	1 (0–2)	0.5 (0–1.75)	1 (0–2)	0 (0–1)	0 (0–1.5)	0 (0–1)	1 (0–2)	0 (0–1.5)	0 (0–1)	1 (0–2)	0 (0–3)
Staff support performance	0 (0–0)	0 (0–0)	0 (0–0)	0 (0–0)	0 (0–0)	0 (0–0)	0 (0–0)	0 (0–0)	0 (0–1)	0 (0–0)	0 (0–0)	0 (0–0)	0 (0–0)
Rating of implementation frequency	2 (2–3)	2 (2–3)	2 (2–3)	2.5 (2–3)	2.5 (2–3)	2.5 (2–3)	2 (2–2.5)	2 (2–3)	2(2–3)	3 (2–3)	2 (2–2.75)	2 (2–3)	2 (2–2.75)

**Table A3:** Description of the VR tasks scores (median (IQR)).

VR Tasks Scores	(Median (IQR))
Attention	98 (88–100)
Range of motion	65 (52–73)
Reaction	66 (43–78)
Holding	84 (57–97)
Accuracy	98 (95–99)
Memory	64 (42–70)

Abbreviations: VR = virtual reality.

**Table A4:** Correlation between PREM items and age, sex, clinical outcomes and VR tasks in pwMS (N = 125) according to Spearman.

	Age	Sex	Disease Duration	FSS Visual	AMSQ	FSS Brainstem	FSS Pyramidal	FSS Cerebellar	FSS Sensory	FSS Cerebral	Ambulation	EDSS Step	VR aROM	VR Attention	VR Holding	VR Reaction	VR Accuracy	VR Memory
**Difficulty**	0.076	0.120	0.101	0.074	**0.224 ***	0.135	**0.188 ***	0.170	**0.174 ***	0.122	**0.215 ***	**0.208 ***	**−0.257 ***	−0.157	**−0.217 ***	−0.127	−0.179	**−0.187 ***
**Self-use of results**	0.078	0.033	−0.095	−0.051	0.030	0.053	−0.026	0.048	−0.105	−0.080	−0.077	−0.033	−0.082	0.008	−0.127	−0.059	**−0.220 ***	−0.147
**Usefulness of the results**	0.118	0.063	−0.080	−0.034	0.040	0.018	−0.035	−0.017	−0.075	−0.147	−0.055	−0.029	**−0.249 ***	0.022	−0.184	−0.046	**−0.243 ***	**−0.188 ***
**Integration of results in therapy**	0.065	−0.071	−0.165	0.015	0.075	−0.101	−0.038	−0.035	−0.145	−0.099	−0.085	−0.104	**−0.230 ***	−0.010	−0.011	−0.033	**−0.297 ***	−0.096
**Acceptance required time**	0.156	0.021	0.041	0.063	0.166	0.049	0.119	0.148	0.076	0.048	0.091	0.122	−0.144	−0.027	**−0.276 ***	−0.153	**−0.196 ***	−0.118
**Strain**	**0.2570***	0.016	0.041	0.064	**0.272 ***	0.162	0.104	0.130	**0.190 ***	0.045	0.123	0.156	−0.065	−0.111	−0.115	−0.073	**−0.287 ***	−0.033
**Safety**	0.034	0.005	−0.103	0.126	0.139	0.017	−0.018	−0.066	0.071	−0.022	−0.043	0.000	−0.049	−0.097	**−0.200 ***	0.007	**−0.228 ***	0.010
**Comfort**	0.160	−0.034	−0.075	0.141	0.149	0.034	0.054	0.008	−0.006	0.011	0.020	0.060	−0.061	−0.015	−0.128	0.006	−0.129	0.008
**Staff support performance**	0.121	0.033	−0.028	0.048	0.073	−0.084	0.005	−0.014	−0.054	0.077	0.008	0.050	0.098	−0.103	−0.113	0.115	−0.093	0.097
**Rating of implementation frequency**	0.064	0.048	−0.005	0.108	0.146	0.107	0.154	**0.191 ***	0.039	0.063	**0.195***	**0.200 ***	**0.272 ***	−0.059	**0.189 ***	0.026	0.064	0.126

* *p* < 0.05, Abbreviations: AMSQ = Arm Function in Multiple Sclerosis Questionnaire, FSS = Functional System Score, EDSS = Expanded Disability Status Scale, VR = virtual reality, aROM = active range of motion.
